# Primary care visits due to mental health problems and use of psychotropic medication during the COVID-19 pandemic in Finnish adolescents and young adults

**DOI:** 10.1186/s13034-023-00584-0

**Published:** 2023-03-09

**Authors:** Ilari Kuitunen, Mikko M. Uimonen, Ville T. Ponkilainen, Ville M. Mattila

**Affiliations:** 1 Institute of Clinical Medicine, University of Eastern Finland, Kuopio, Finland; 2 Department of Pediatrics, Mikkeli Central Hospital, Porrassalmenkatu 35-37, 50100 Mikkeli, Finland; 3 Central Finland Hospital Nova, Jyväskylä, Finland; 4 Faculty of Medicine and Health Technologies, Tampere University, Tampere, Finland; 5 Tampere University Hospital, Tampere, Finland

**Keywords:** COVID-19, Mental health, Psychotropic medication, Epidemiology, Adolescence

## Abstract

**Background:**

Social restrictions due to COVID-19 have impacted the everyday life of adolescents and young adults, with increased levels of stress and anxiety being reported. Therefore, we report primary care visits due to mental health problems and the use of psychotropic medication in Finland.

**Methods:**

We conducted a nationwide register-based study and included primary care visits with mental health problems (F*-class ICD-10 diagnosis) for patients aged 15–24 years. We calculated incidence for visits and used incidence rate ratios (IRR) for comparisons. Psychotropic medication purchases for patients aged 13–24 years were included. Annual psychotropic medication user prevalence per 1000 was calculated and prevalence rate ratios (PRR) with 95% confidence intervals (CI) were used for comparisons. The years 2020 and 2021 were compared to the pre-pandemic reference year 2019.

**Results:**

A total of 396534 visits to primary care due to mental health problems were included. Annual visit incidences per 1000 were 151.7 in 2019, 193.6 in 2020, and 306.7 in 2021, indicating a 28% (IRR 1.28, CI 1.27–1.29) increase from 2019 to 2020 and a 102% (IRR 2.02, CI:2.01–2.04) increase from 2019 to 2021. Highest reported increases in 2020 were sleeping disorders (IRR 1.79, CI 1.72–1.87) and anxiety disorders (IRR 1.39, CI 1.37–1.42). Prevalence of antidepressant use increased by 25% (PRR 1.25, CI 1.23–1.26) in 2021. An increase was also seen in the use of antipsychotics (+ 19%, PRR 1.19. CI 1.16–1.21).

**Conclusions:**

The COVID-19 pandemic increased the need for mental health services and medication among Finnish adolescents and young adults. Our health care system needs the capacity to manage the increased number of visits, and we must be better prepared for future crises.

**Supplementary Information:**

The online version contains supplementary material available at 10.1186/s13034-023-00584-0.

## Introduction

After the outbreak of the COVID-19 pandemic, societal restrictions aimed at constraining the spread of the disease, including the closing of schools and other educational facilities as well as recommendations for telecommuting, reduced daily social interactions. Moreover, the prolonged pandemic also led to permanent social deprivation which, in turn, has raised concerns about mental well-being, especially in adolescence and early adulthood [1–5]. Uncertainty about the future in general and anxiety about the lack of economic stability caused by the risk of unemployment and precarious societal situation has only made the problem worse. Indeed, mental health problems are known to increase in the wake of societal and economic crises [6–8]. Concurrently with the increasing burden of psychosocial distress caused by government-imposed lockdowns, the availability of mental health services decreased in spring 2020, leading to a reduction in emergency department visits by individuals with mental health problems [9]. This reduction in services created ideal circumstances for an increase in mental health problems and a future mental health treatment burden was predicted [10]. The cost of protecting the most vulnerable in society against the COVID-19 disease was that young people living a stage of their life related to strong social dependency and subsequent breakthrough to the labor market were predisposed to psychosocial crisis. Indeed, previous studies have shown that younger populations were affected more by the lockdown mentally than older populations [11].

In Finland, the prevalence of mental health problems has been reported to be relatively high when compared to the global average [12]. Although the incidence of COVID-19 infections was relatively low in Finland, extensive restrictions and public facility lockdowns were implemented in March 2020 [13]. These restrictions included school closures, restriction of gatherings and closure of restaurants and bars. Since May 2020, restrictions on the activities (schools and hobbies) of young children have been avoided, but adolescents (aged 15 or older) and young adults have had to endure continuous remote learning periods and restrictions on their hobbies and activities, and also restaurants and bars have been closed early. Overall social encounters have been restricted temporarily for adolescents and young adults in spring 2020, fall and winter 2020–2021 and again in fall 2021 [14].

There are only few studies that have examined the mental health in children and adolescents in Finland during the pandemic. A brief report from Finland found that the prevalence of antidepressant users increased during the pandemic in children aged 6 to 12 years [15]. Another study from Finland described how the use of remote visits did not reduce the visit rates in specialized adolescent unit in Finland [16]. However, national estimates in visit rates due to mental health problems and psychotropic medication consumption in adolescents in Finland are lacking. Thus, we aim to evaluate how the COVID-19 pandemic and related societal restrictions affected the mental wellbeing of adolescents and young adults in Finland.

## Methods

We conducted a nationwide register-based retrospective surveillance study from 2019 to 2021. Data were gathered from three open-access registers. The number of primary health care visits to physicians due to mental health problems was collected from the Care Register for Primary Health Care, which is maintained by the Finnish Institute of Health and Welfare. The register has excellent coverage, as over 90% of the Finnish primary care centers report data to it [17]. Visits with mental health related F category (mental and behavioral disorders) diagnostic codes (International Classification of Diseases 10^th^ version) were included (Additional file 1: Table S1). Based on these diagnoses, we calculated the yearly incidence per 1000 adolescents and young adults aged 15–24 years in primary care due to mental health problems. The age group is pre-stratified by the Finnish Institute of Health and Welfare (registry owner) for the open-access data. As we defined the inclusion based on the diagnostic code (F-class) we do not have missing information on visit rates. Visits without F-class diagnoses were thus all excluded. One visit may have more than one diagnose, but all of the diagnoses should be relevant to the visit and describe the visit.

In Finland mental health problems are treated in primary care. The patient first meets either a physician or nurse who is specialized to mental health. Prescriptions and medication decisions are made by physicians and similarly sick leave is prescribed physicians only. Severe or treatment persistent cases are referred from primary care to specialized psychiatric healthcare (secondary or tertiary level units with outpatient clinics). Some larger primary care centers have own specialized psychiatrics hired to reduce the need to referrals to specialized healthcare, but these practices have large variations between cities.

In addition, we collected all psychotropic medications prescribed by a physician from the Register of Reimbursable Medicine Costs, which is maintained by the Social Insurance Institution of Finland. Finland has a universal tax-funded health care system, where all medication purchases are reported to the register regardless of the setting of the prescription (primary care, specialized care, hospitals, and private clinics) [18, 19]. The register does not, however, contain information on dosage, indication, or for how long the medication was prescribed. Therefore, we calculated the prevalence of medication users per 1000, and labeled persons as users if they purchased medication from the pharmacy. One person might have purchased several different classes of medication. We have included the medications based on the Anatomical Therapeutic Chemical (ATC) Classification system (Additional file 1: Table S2). We analyzed the main classes and the most used medications more specifically. As the register uses default age stratification (which is defined by the register holder), we included medication information for all adolescents aged 13 to 24 years.

Finally, we gathered the population size for each age group from the Population Information System at the end of the year in question and used it as the denominator in the incidence and prevalence calculations [20]. Incidence and prevalence were calculated per 1000 persons per age-group with 95% confidence intervals (CI). Incidence comparisons between the pandemic years (2020 and 2021) and the reference year (2019) were made by incidence rate ratios (IRR), and prevalence comparisons were made by prevalence rate ratios (PRR) with CI.

As we used open-access data, no research permissions or ethical committee evaluations were required. All the data generated in this process have been provided as an appendix (Additional file 2: Table S2).

## Results

A total of 396534 visits with ICD-10 F-diagnoses to primary health care were included. Of these, 92609 (23.3%) occurred in 2019, 117459 (29.6%) in 2020, and 186466 (47.1%) in 2021. Respective annual visit incidences per 1000 were 151.7, 193.6, and 306.7, indicating a 28% (IRR 1.28, CI 1.27–1.29) increase from 2019 to 2020 and a 102% (IRR 2.02, CI 2.01–2.04) increase from 2019 to 2021 (Table 1).

**Table 1:** Number and incidence per 1000 adolescents of visits to primary care stratified by diagnoses

ICD-10^a^	2019		2020			2021		
	n	Incidence	n	Incidence	IRR (95% CI)	n	Incidence	IRR (95% CI)
F10-F19 Mental and behavioral disorders due to psychoactive substance use	7967	13.0	6198	10.2	0.78 (0.76–0.81)	6880	11.3	0.87 (0.84–0.90)
F10 Alcohol	1185	1.9	1445	2.4	1.23 (1.14–1.33)	1570	2.6	1.33 (1.23–1.43)
F20-F29 Schizophrenia, schizotypal and delusional disorders	2108	3.5	2652	4.4	1.27 (1.20–1.34)	2456	4.0	1.17 (1.10–1.24)
F30-F39 Mood [affective] disorders	32,210	52.8	39,145	64.5	1.22 (1.21–1.24)	59,997	98.7	1.87 (1.85–1.90)
F31 Bipolar affective disorder	1209	2.0	1650	2.7	1.37 (1.28–1.48)	2431	4.0	2.02 (1.88–2.16)
F32 Depressive episode	25,005	41.0	29,576	48.8	1.19 (1.17–1.21)	45,898	75.5	1.84 (1.82–1.87)
F33 Recurrent depressive disorder	4503	7.4	5970	9.8	1.33 (1.28–1.39)	8968	14.8	2.00 (1.93–2.07)
F34 Persistent mood [affective] disorders	1035	1.7	1298	2.1	1.26 (1.16–1.37)	1636	2.7	1.59 (1.47–1.72)
F40-F45 Neurotic, stress-related and somatoform disorders	44,038	72.1	59,487	98.1	1.36 (1.34–1.38)	100175	164.8	2.28 (2.26–2.31)
F40 Phobic anxiety disorders	4826	7.9	5713	9.4	1.19 (1.15–1.24)	8593	14.1	1.79 (1.73–1.85)
F41 Other anxiety disorders	29,007	47.5	40,176	66.2	1.39 (1.37–1.42)	70,728	116.3	2.45 (2.42–2.48)
F42 Obsessive–compulsive disorder	2263	3.7	3026	5.0	1.35 (1.27–1.42)	4933	8.1	2.19 (2.08–2.30)
F43 Reaction to severe stress, and adjustment disorders	4040	6.6	6490	10.7	1.62 (1.55–1.68)	12,989	21.4	3.23 (3.12–3.35)
F45 Somatoform disorders	3634	6.0	3685	6.1	1.02 (0.97–1.07)	2350	3.9	0.65 (0.62–0.68)
F50 Eating disorders	2503	4.1	3241	5.3	1.30 (1.24–1.37)	5133	8.4	2.06 (1.96–2.16)
F51 Nonorganic sleep disorders	3783	6.2	6736	11.1	1.79 (1.72–1.87)	11,825	19.5	3.24 (3.03–3.26)
Total combined	92,609	151.7	117459	193.6	1.28 (1.27–1.29)	186466	306.7	2.02 (2.01–2.04)

Pandemic years 2020 and 2021 compared to reference year 2019 by incidence rate ratios (IRR) with 95% confidence intervals (CI)

^a^ICD-10 = International Classification of Diseases 10th version

**Fig. 1:**
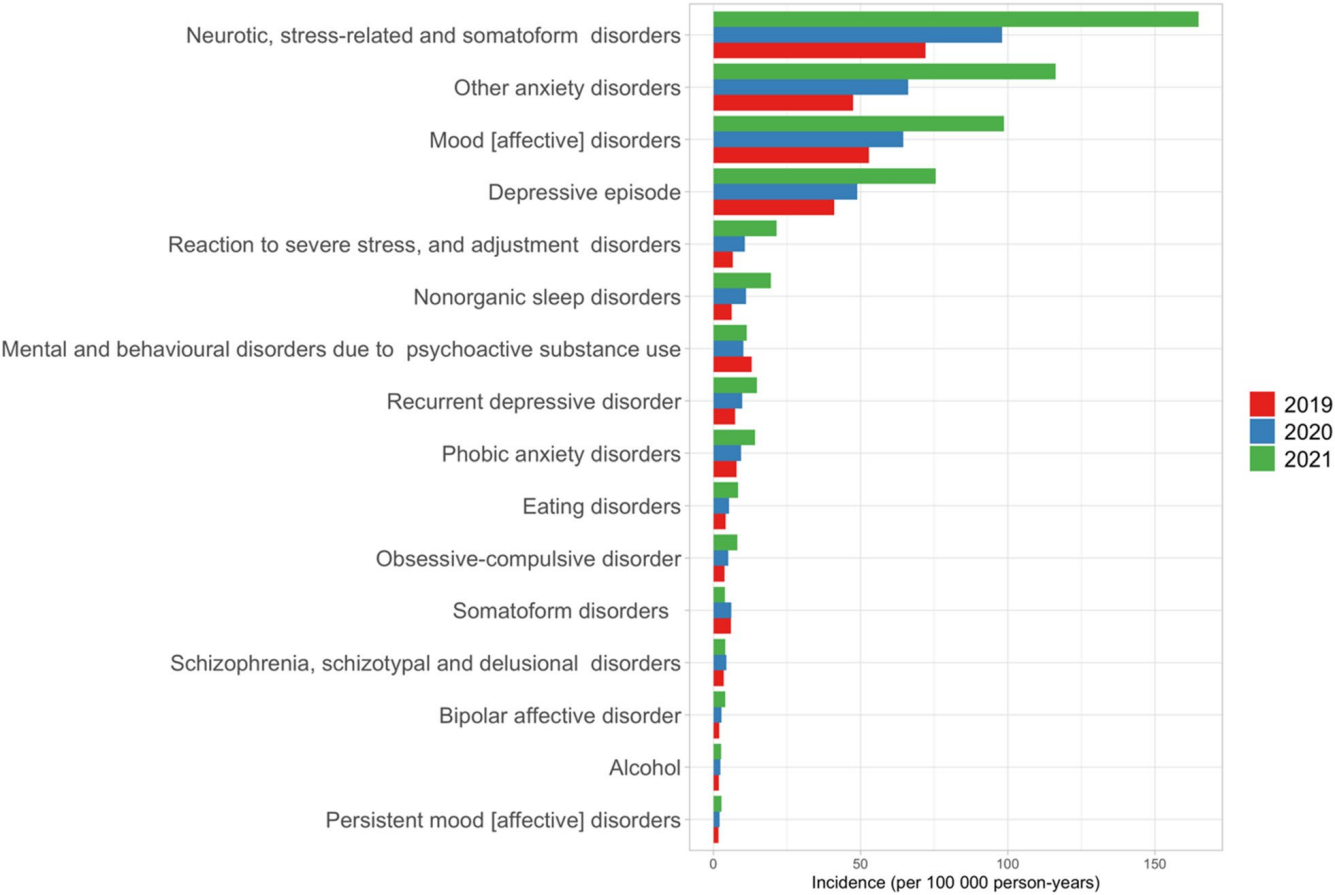
The incidence of primary health care visits due to mental health problems in adolescents and young adults (aged 15–24 years) from 2019 to 2021 in Finland. Footnote: All differences were statistically significant, except the change in somatoform disorders in 2020

The majority of the visits were due to neurotic, stress-related and somatoform disorders (F40-F45), and mood disorders (F30-F39). The highest reported increases in 2020 were due to sleeping disorders (+ 79%, IRR 1.79, CI: 1.72–1.87) and anxiety disorders (+ 39%, IRR 1.39, CI 1.37–1.42). When compared to 2019, the highest increases in 2021 were in visits due to sleeping disorders (+ 224%, IRR 3.24, CI 3.12–3.35) and anxiety disorders (+ 128%, IRR 2.45, CI 2.42–2.48). The visit rate due to eating disorders had more than a two-fold increase. The increase in visit incidences was seen in all diagnostic categories except for visits due to substance abuse in 2021 (Fig. 1).

Psychotropic medication user prevalence increased from 2019 to 2020 and 2021. In 2020, the highest reported increase was in the prevalence of vortioxetine users (+ 23%, PRR 1.23, CI 1.17–1.29) and sertraline users (+ 20%, PRR 1.20, CI 1.12–1.23) (Table 2). Anxiolytics user prevalence decreased by 4% (PRR 0.96, CI 0.93–0.99) in 2020. In 2021, the highest reported prevalence increase was in the users of vortioxetine (+ 65%, PRR 1.65, CI 1.57–1.73) and sertraline (+ 53%, PRR 1.53, CI 1.49–1.57). Overall, the prevalence of antidepressant use increased by 25% (PRR 1.25, CI 1.23–1.26). An increase was also seen in the use of antipsychotics (PRR 1.19, CI 1.16–1.21). However, the prevalence of hypnotics and sedatives users decreased by 10% (PRR 0.90, CI 0.85–0.94) during this period (Table 2).

**Table 2:** Number and prevalence (per 1000) of psychiatric medication users in the pandemic years 2020 and 2021 compared to reference year 2019

ATC = Anatomical therapeutical classification	2019		2020			2021		
	n	Prevalence	n	Prevalence	PRR (CI)	n	Prevalence	PRR (CI)
N05A Antipsychotics	21,824	29.8	23,581	32.3	**1.08 (1.06–1.10)**	25,876	35.3	**1.19 (1.16–1.21)**
N05AH02 Clozapine	575	0.8	557	0.8	0.97 (0.84–1.09)	585	0.8	1.02 (0.93–1.14)
N05AH03 Olanzapine	3049	4.2	3454	4.7	**1.14 (1.08–1.19)**	3710	5.1	**1.22 (1.16–1.28)**
N05AH04 Quetiapine	14809	20.2	16,132	22.1	**1.09 (1.07–1.12)**	17,713	24.2	**1.20 (1.17–1.22)**
N05An01 Lithium	416	0.6	456	0.6	1.10 (0.96–1.26)	495	0.7	**1.19 (1.04–1.36)**
N05AX08 Risperidone	4118	5.6	4276	5.9	1.04 (1.00–1.09)	4571	6.2	**1.11 (1.06–1.16)**
N05AX12 Aripripratzol	3246	4.4	3 696	5.1	**1.14 (1.09–1.20)**	4266	5.8	**1.31 (1.26–1.38)**
N05B Anxiolytics	7823	10.7	7502	10.3	**0.96 (0.93–0.99)**	7882	10.8	1.01 (0.98–1.04)
N05BA01 Diazepam	437	0.6	430	0.6	0.99 (0.86–1.13)	419	0.6	0.96 (0.84–1.10)
N05BA04 Oxazepam	6262	8.5	6048	8.3	0.97 (0.94–1.00)	6457	8.8	1.03 (1.00–1.07)
N05BA09 Clonazepam	426	0.6	431	0.6	1.01 (1.11–1.16)	457	0.6	1.07 (0.94–1.22)
N05BA12 Alprazolam	617	0.8	513	0.7	**0.83 (0.74–0.94)**	445	0.6	**0.72 (0.64–0.81)**
N05C Hypnotics and sedatives	3632	5.0	3572	4.9	0.99 (0.94–1.03)	3254	4.4	**0.90 (0.85–0.94)**
N05CD08 Midazolam	1070	1.5	1153	1.6	1.08 (0.99–1.17)	942	1.3	**0.88 (0.81–0.96)**
N05CF01 Zopiclone	1568	2.1	1515	2.1	0.97 (0.90–1.04)	1447	2.0	**0.92 (0.86–0.99)**
N05CF02 Zolpidem	1059	1.4	985	1.3	0.93 (0.86–1.02)	926	1.3	**0.87 (0.80–0.96)**
N06A Antidepressants	48,273	65.9	51,853	71.0	**1.08 (1.06–1.09)**	60,263	82.3	**1.25 (1.23–1.26)**
N06AB Selective serotonin reuptake inhibitors	35,161	48.0	37,772	51.7	**1.08 (1.06–1.09)**	44,560	60.8	**1.27 (1.25–1.29)**
N06AB03 Fluoxetine	9484	12.9	10,143	13.9	**1.07 (1.04–1.10)**	12,189	16.6	**1.29 (1.25–1.32)**
N06AB04 Citalopram	2394	3.3	2131	2.9	**0.89 (0.84–0.95)**	1976	2.7	**0.83 (0.78–0.88)**
N06AB05 Paroxetine	693	0.9	708	1.0	1.02 (0.92–1.14)	797	1.1	**1.15 (1.04–1.27)**
N06AB06 Sertraline	9582	13.1	11,474	15.7	**1.20 (1.17–1.23)**	14,623	20.0	**1.53 (1.49–1.57)**
N06AB10 Escitalopram	15,237	20.8	15,630	21.4	**1.03 (1.01–1.05)**	17,960	24.5	**1.18 (1.15–1.20)**
N06AX Other antidepressants	19,950	27.2	21,615	29.6	**1.09 (1.07–1.11)**	24,875	34.0	**1.25 (1.22–1.27)**
N06AX11 Mirtazapine	9235	12.6	10,114	13.8	**1.10 (1.07–1.13)**	11,329	15.5	**1.23 (1.19–1.25)**
N06AX12 Bupropion	3087	4.2	3443	4.7	**1.12 (1.07–1.17)**	4322	5.9	**1.40 (1.34–1.47)**
N06AX16 Venlafaxine	5866	8.0	5882	8.1	1.01 (0.97–1.04)	6459	8.8	**1.10 (1.06–1.14)**
N06AX21 Duloxetine	1614	2.2	1663	2.3	1.03 (0.97–1.11)	1849	2.5	**1.15 (1.07–1.23)**
N06AX26 Vortioxetine	2944	4.0	3611	4.9	**1.23 (1.17–1.29)**	4851	6.6	**1.65 (1.57–1.73)**

Medication user defined as dispensed at least one medication from a pharmacy during the year. Comparisons made by prevalence rate ratios (PRR) with 95% confidence intervals (CI). Psychiatric medication stratified based on the anatomic therapeutic chemical (ATC) classification system. Statistically different findings have been bolded

## Discussion

The main finding of our study was that the incidence of primary health care visits due to mental health problems doubled from the year prior to the start of the COVID-19 pandemic (2019) to the year after the start of the pandemic (2021). The incidence of visits also increased during the first year of the COVID pandemic (2020), even though non-emergent visits were reduced to ensure there were enough resources to treat COVID-19 patients. There have been reports that patients with mental health problems experienced restricted health care services during the pandemic [21]. Therefore, it may be that despite the increasing incidence, many of the planned or needed visits were canceled. Therefore, our data may underestimate the true incidence in 2020.

Most worryingly, the biggest increase in the reasons for primary health care visit were stress-related, anxiety, and depressive disorders, in addition to sleeping and eating disorders. This increase can be explained by the uncertain future in general and the lack of economic stability due to the risk of unemployment and the precarious societal situation. As primary health care is the first point of health care contact for most Finnish adolescents, these changes reflect the prevalence of mental health issues in the general population. Despite the worrying change, it is interesting that primary health care resources have been sufficient to handle double the incidence of visits. In addition, the decreased incidence of mental disorders due to substance use is of course a welcomed change. However, this may merely reflect a decrease in the number of social events. Unfortunately, visits to primary health care due to alcohol abuse increased in 2020 and 2021, a finding which has been supported previously in Finnish questionnaire surveillance studies and emergency department studies on alcohol intoxication in Finnish adolescents [22–25]. Compared to other countries the decrease in the substance use was rather surprising as a previous meta-analysis suggested that substance use and alcohol use increased during the pandemic [26].

In the present study, we observed an increase in the prevalence of patients using antidepressants and antipsychotics, whereas the prevalence of patients using anxiolytics, hypnotics, and sedatives decreased from the level in the year before the COVID-19 pandemic. Therefore, it seems that in addition to an increase in symptoms leading to the first contact with health care services, the number of patients in need of treatment has also increased. Similar findings of increased antidepressant use have been reported globally [27–29]. Moreover, the use of antidepressants has increased more in younger patients than in the older population [30, 31]. A Canadian study found that the use of psychotropic medications decreased overall during the first three months of the pandemic, after which the incidence of antidepressant use increased notably [32] The same Canadian study also found that the use of anxiolytics continued the decreasing trend which started prior to pandemic [32] Similar decrease was seen in our current study. Interestingly a Danish study conducted in adolescents found that the consumption of psychotropic medication increased in all classes except anxiolytics [33]. We had similar findings regarding the anxiolytic consumption in our study, but contraindicatory we did not find evidence of increased use of hypnotics and sedatives. Due to the limitations of open-access data, we were unable to investigate whether the number of psychotherapy appointments had also increased, as the increased number of prescriptions for medication can either mean patients are receiving treatment more often or that the availability of other treatments has become more limited.

In Finland, the impact of the COVID-19 pandemic on the mental health of adolescents and young adults has only been studied among students in higher education [34] In their study, Sarasjärvi et al. Noted that academic stress was associated more with mental health than COVID-19 itself. Globally, it has become clear that adolescents and young adults especially have been affected the most by the restrictions, and therefore have an increased need for mental health services compared to the pre-pandemic situation [11, 31, 35].

The strength of this study is that we used multiple high-quality nationwide registers that contain most of the primary health care visits. Furthermore, the medication data includes all prescriptions, and thus we have a nationwide sample of over 700 000 adolescents and young adults. The limitations of this study are that the data was age-grouped by the register holder and was annual instead of monthly statistics. Thus, we were unable to study age-groups or monthly variations more thoroughly. A second limitation is a lack of information from the private sector and Finnish University Healthcare Services, as this data is not publicly available. However, all university students are eligible to use primary care services and the medication prescriptions from the private sector and University Healthcare Services are included in our study. We can, therefore, estimate the true prevalence of medication users. A minor limitation is the lack of information whether the visit was an in-person or a telehealth visit, as the register does not contain this information. A further limitation is the lack of more precise information on patients, such as socioeconomic status or history of previous medications, gender, and education as these would have helped better estimate what factors influenced most to the increased burden of mental health problems during the pandemic. Final limitation is the lack of precise prescription information and though a defined daily dose consumption changes could not have been estimated.

## Conclusion

The incidence of primary health care visits due to mental health problems increased rapidly after the beginning of the COVID-19 pandemic. The largest increase in the reasons for the visits were stress-related disorders, anxiety and depression, and sleeping and eating disorders. The COVID-19 pandemic has increased the need for mental health services and medication among Finnish adolescents and young adults. Our health care system needs the capacity to manage the increased number of visits, and we must learn from this experience and be better prepared for future crises.

## Supplementary Information

**Additional file 1: Table S1.** Included diagnostic codes based on the ICD-10 (International Classification of Diseases 10th version) and the grouping used in our study based on the diagnoses. **Table S2.** Included medicines based on the ATC (Anatomical therapeutic classification)

**Additional file 2.** Annual number of visits stratified by ICD-10 diagnoses.

## Data Availability

Available per request from corresponding author.
