# Supplementary material for: Primary care visits due to mental health problems and use of psychotropic medication during the COVID-19 pandemic in Finnish adolescents and young adults
Source: Child Adolesc Psychiatry Ment Health. 2023 Mar 9;17:35. doi: 10.1186/s13034-023-00584-0 (PMC9998142; doi:10.1186/s13034-023-00584-0)
Supplement: Supplementary file 1 — Additional file 1: Table S1. Included diagnostic codes based on the ICD-10 (International Classification of Diseases 10th version) and the grouping used in our study based on the diagnoses. Table S2. Included medicines based on the ATC (Anatomical therapeutic classification) [file 13034_2023_584_MOESM1_ESM.docx]

**Additional file materials**

**Additional file 1: Table S1** Included diagnostic codes based on the ICD-10 (International Classification of Diseases 10^th^ version) and the grouping used in our study based on the diagnoses.

| ICD-10 | Explanation |
| --- | --- |
| F10 – F19 | Mental and behavioral disorders due to psychoactive substance use |
| F20 – F29 | Schizophrenia, schizotypal and delusional disorders |
| F30 – F39 | Mood [affective] disorders |
| F40 – F45 | Neurotic, stress-related and somatoform disorders |
| F50 | Eating disorders |
| F51 | Sleeping disorders |

**Additional file 1: Table S2** Included medicines based on the ATC (Anatomical therapeutic classification)

| ATC | Explanation |
| --- | --- |
| N05A* | Antipsychotics |
| N05B* | Anxiolytics |
| N05C* | Hypnotics and sedatives |
| N06A* | Antidepressants |
